# Stigmatization and psychological impact of COVID-19 pandemic on frontline healthcare Workers in Nigeria: a qualitative study

**DOI:** 10.1186/s12888-021-03540-4

**Published:** 2021-10-20

**Authors:** Ayi Vandi Kwaghe, Vivian Gga Kwaghe, Zaiyad Garba Habib, Gga Vandi Kwaghe, Olayinka Stephen Ilesanmi, Bissalah Ahmed Ekele, Chukwuma David Umeokonkwo, Muhammad Shakir Balogun

**Affiliations:** 1 Nigeria Field Epidemiology and Laboratory Training Programme, Abuja, Nigeria; 2 Department of Veterinary and Pest Control Services, Federal Ministry of Agriculture and Rural Development, Abuja, Nigeria; 3 Department of Internal Medicine, University of Abuja Teaching Hospital, Gwagwalada, Abuja, Nigeria; 4 Department of Obstetrics and Gynaecology, University of Abuja Teaching Hospital, Gwagwalada, Abuja, Nigeria; 5 Department of Community Medicine, College of Medicine, University of Ibadan and University College Hospital, Ibadan, Oyo State Nigeria; 6 Department of Community Medicine, Alex Ekwueme Federal University Teaching Hospital Abakaliki, Abakaliki, Ebonyi State Nigeria

**Keywords:** Coronavirus, COVID-19, Mental health, Stigmatization, Frontline healthcare workers, COVID-19 isolation Centre, Nigeria

## Abstract

**Introduction:**

The COVID-19 pandemic has had a great toll on global health. Frontline healthcare workers (FHCW) directly involved in the treatment of COVID-19 patients have faced some physical and psychological challenges. This study explored the stigma and traumatic experiences of the FHCW during the COVID-19 pandemic in Nigeria.

**Methods:**

We recruited twenty FHCW directly involved in the treatment of COVID-19 patients through purposive and snowball sampling techniques. Face-to-face in-depth interviews were conducted for all participants, and qualitative analysis of data was done using Colaizzi’s phenomenological method.

**Results:**

Five themes identified were: Early stage of the pandemic (fear, anxiety, public fright, other countries repatriating their citizens, the socio-economic impact of the pandemic and a call to duty for the FHCW); working with COVID-19 patients (excitement on patients recovery and duty stress); psychological, mental and emotional trauma; stigmatization (stigmatized by colleagues, family, friends or their residential communities, reasons for stigmatization which were fear of infection, limited knowledge of the virus and working at the isolation centre and the effect of stigma); and recommendations (education and awareness creation, government showing more care towards the FHCW and provision of health insurance for FHCW to take care of those that get infected in the line of duty).

**Conclusion:**

Stigmatization has proven to be a major challenge for FHCW in conducting their duties. The psychological impact experienced by FHCW may affect the quality of the services rendered by these workers. The study reveals the need of education and awareness creation in the ongoing pandemic. There is a need for the government and society to acknowledge and appreciate the efforts of FHCW.

## Background

Nigeria is multi-ethnic with diverse cultures due to the various ethnic groups. The major ethnic groups are the Hausas based in the north, the Yoruba’s in the southwest and the Igbos in the southeastern part of the country with Christianity and Islam as major religions in the country [1]. The country has rich human resource with a population of about 202 million people, has one of the largest youth population in the world, an abundance of natural resources, the biggest oil exporter and has the largest natural gas reserves in Africa [2].

Nigeria was not left out of the global economic disruption; the economy of the nation depends mainly on the oil sector which made the nation’s economy vulnerable with the advent of the C0VID-19 pandemic as a result of the massive decline of oil price during the pandemic [3]. Furthermore, eighty three million Nigerians accounting for 40% of the population live below the poverty line, 53 million (25%) are vulnerable to poverty and with the ongoing COVID-19 pandemic, many of these 53 million vulnerable people are liable to fall into poverty [3]. The high inflation and unemployment exacerbate macroeconomic risks, and activity in the tertiary sector will not fully normalize unless the pandemic is contained [3].

Public Health is relevant to national security, despite the huge development in the health care sector there is need for improvement as shown by the various health indicators in the nation. Challenges faced by the sector such as financial managerial competency, inadequate funding and limited human resources is likely to increase the impact of the pandemic [4–6]. The impact of the pandemic on health will be dependent on the duration and spread of the pandemic [3].

Abuja, the capital city of Nigeria and has the second highest number of cases in the first wave of the COVID-19 pandemic [7]. Despite the challenges and hitches that arose in the health sector as a result of the pandemic, the health sector was able to handle the situation with the help of the government and other nongovernmental organisations that supported the health sector with PPEs and other equipment’s [8, 9]. Frontline healthcare workers (FHCW) are at highest risk of being infected. These are people working in health institutions providing direct care and treatment services in order to save lives in their communities [10]. The duties of FHCW in the face of COVID-19 pandemic has taken its toll on them considering the fact that there are many unknown variables regarding the novel corona virus infection. Stigmatization is one of the major issues associated with the highly infectious novel corona virus pandemic [11–13]. Among the challenges faced by these frontline healthcare workers are stigmatization, resulting in psychological and emotional trauma on the person that is being stigmatized [14].

Stigma has a high impact on healthcare workers outcomes [15]. Working with patients that possess the potential of being highly infectious such as the COIVD-19 pandemic serves as a means by which stigma is generated [16]. Several studies have indicated that stigma associated with COVID-19 is a major source of psychological trauma resulting in stress, anxiety and depression among FHCW with adverse health implications [17–21]. A systemic review with meta-analysis revealed that all the articles studied reported on stigmatization as a result of work-related COVID-19 exposure [22]. Studies have revealed a high prevalence of stress, depression, anxiety and mental disorders among FHCW in the COVID-19 pandemic [23]. There exist a global barrier to health seeking behaviour as a consequence of stigmatization [24] propelling diverse forms of discrimination mostly resulting in reduced or absence of social acceptance or opportunities to specific individuals or groups of people. Moral injury is a product of intense psychological distress from actions or inactions violating an individual’s moral or ethical code [25, 26]. The advent of potentially morally injurious events may consequently produce negative thoughts about oneself or others along with deep feelings of shame, guilt or disgust [27]. Consequently attributing to the development of mental health problems such as depression, post-traumatic stress disorder and anxiety [27, 28] The FHCW trying to save lives and protect society may also experience social distancing, changes in the behavior of family members, and stigmatization for being suspected of having COVID-19 [29].

Qualitative methods are most effective in capturing social responses to the COVID-19 pandemic as shown by other epidemics in the health sector [30, 31]. Such methods permits the capturing and comprehension of how people make meaning and sense of health and illness [30, 31]. Despite the struggles of FHCW in the COVID-19 pandemic in Nigeria, there is no psychological counselling readily available for the FHCW who are faced with the challenges of increased work stress, depression, anxiety, stigmatization and mental trauma. There is need for instituting measures to handle these challenges. This study explored the experiences of the FHCW during the COVID-19 pandemic in Nigeria.

## Method

### The aim, design and setting of the study

The University of Abuja Teaching Hospital (UATH) COVID-19 isolation centre is located in Gwagwalada. It is one of the seven isolation centres in the Federal Capital Territory (FCT). The centre is responsible for handling patients that have moderate to severe symptoms of COVID-19 infections in the FCT. It is a 42 bedded capacity isolation centre and was the first hospital in the FCT to manage patients with COVID-19. Majority of cases from this centre were referred from other isolation centres in the FCT for specialist care.

We aimed at determining the challenges faced by the FHCW in Nigeria in the COVID-19 pandemic; there psychological and mental state and their means of coping in the face of the pandemic. The research questions were: Are FHCW in Nigeria battling with stigmatization and psychological trauma? What are the measures that could help in resolving these challenges?

We conducted a qualitative study on FHCW at the UATH isolation centre, Gwagwalada, Abuja. The inclusion criterion was FHCW working at the UATH isolation centre, Gwagwalada. The exclusion criteria were inability to participate in two or more interviews by a person within the study period. Purposive and snowball sampling were used in the recruitment of participants. We conducted the study from the 14th to 29th September, 2020 and determined the data “saturation point”; when there was no new information generated from the interview. Duration of interviews ranged from 5:48 to 19:47 min with an average interview time of 10:42 min. Twenty FHCW working at the isolation centre were interviewed (five doctors, six nurses, four laboratory scientists and 5 hygienists). Face-to-face in-depth interviews for all participants was used to collect data. We adopted this interview method for the participants’ privacy, confidentiality, exploration of individual views and provision of in-depth information. This method was also more suitable for sensitive issues such as stigmatization and mental health challenges in the face of the ongoing COVID-19 pandemic. The public health advisory on physical distancing, use of face mask and hand hygiene were observed throughout the interviews. We used a SONY_®_ Stereo IC Recorder ICD-PX470 to record all the interviews which were conducted in English language. Obtained recording was transcribed verbatim to effectively communicate the experiences of respondents.

### Interviewer guide

We used an interviewer guide to keep to the scope of the study and ensured that the same stream of guided questions was responded to by all respondents. Questions used for the study were adapted from previous studies [14, 32] and modified to suit the current study. All authors reviewed and approved the interviewer guide. The guide covered areas like: 1. Introduction of the interviewer. 2. Sociodemographic characteristic of the respondent (age, sex, marital status and occupation). 3. What are your insights in the face of the COVID-19 pandemic [32]? along with further probing questions (a) Please tell me your perception generally about the COVID-19 pandemic? (b) How did you feel when you heard about the global impact of the disease and when it finally reached Abuja, the Federal Capital Territory of Nigeria? (c) How did you feel when accepting the COVID-19 outbreak task [32]? (d) How do you feel when working with COVID-19 patients [32]? 4. What are your thoughts and feelings regarding this task [32]? 5. What are your challenges so far regarding your mental health as a frontline health worker? 6. Did the COVID-19 pandemic affect the performance of your duty as a frontline healthcare worker in any way? 7. Do you feel stigmatized in any way [14]? (a) Any form of stigmatization by family members [14]? (b) Have you experienced any form of stigmatization by your colleagues [14]? (c) Did you observe any form of stigmatization by your community [14]? 8. Any idea or reasons for the stigmatization? 9. What are the measures that can be taken to alleviate what you are passing through [14]? 10. Do you have health insurance? Probing questions like “Can you please throw more light on that?”, “Can you please explain?”, “Please tell us more?” were used to enhance the depth of discussion.

### Data analysis

We carried out qualitative analysis of our data using the Colaizzi’s phenomenological method [33]. We analyzed our data using the seven vital steps of descriptive phenomenon while sticking to the content of our data by familiarizing ourselves with the data through identifying significant statements with direct relevance to the phenomenon under investigation, formulating meanings, clustering of themes, developing of exhaustive description of the phenomenon incorporating all the themes produced, producing the fundamental structure and finally, sought verification of the fundamental structure by some of the participants’ [33]. Participants that verified the fundamental structure of the analysed data approved of the findings. The interview data set was manually coded using inductive coding method [34]. Initial response and observation was used to construct a coding scheme based on the major categories that evolved [34].

## Results

The demographic characteristics of respondents on sex, marital status and identification is shown in Table 1.

**Table 1:** Demographic characteristics of respondents

s/no	Sex	Marital Status	Occupation	Identification
1.	Male	Married	Medical Doctor	M1
2.	Female	Married	Medical Doctor	M2
3.	Male	Married	Medical Doctor	M3
4.	Male	Married	Medical Doctor	M4
5.	Male	Married	Medical Doctor	M5
6.	Male	Married	Nurse	N1
7.	Male	Married	Nurse	N2
8.	Female	Single	Nurse	N3
9.	Male	Married	Nurse	N4
10.	Male	Married	Nurse	N5
11.	Male	Married	Nurse	N6
12.	Male	Married	Laboratory Scientist	L1
13	Male	Married	Laboratory Scientist	L2
14	Male	Married	Laboratory Scientist	L3
15	Male	Married	Laboratory Scientist	L4
16	Male	Married	Hygienist	H1
17	Male	Single	Hygienist	H2
18	Male	Single	Hygienist	H3
19	Male	Single	Hygienist	H4
20	Male	Married	Hygienist	H5

### Theme one: early stage of the pandemic

The early phase of the pandemic was characterized by anxiety, socio-economic impact, a call to duty for the FHCW and the need for more engagement by the government in the fight against the pandemic (Table 2).

**Table 2:** Themes, subthemes, codes and frequency of codes mentioned by participants

Themes	Subthemes	Codes	Frequency of codes mentioned by participants
Early phase of the pandemic	Effects of the pandemic	Fear and anxiety	(M2)*3/(M3)*16/(M5)*6/(N1)*2/(N3)*1/(N4)*5/(N5)*2/(N6)*1/(L1)*3/(L2)*8/(L3)*6/(L4)*6/(H1)*2/(H2)*2/(H3)*5/(H4)*1/(H5)*4
		Global, social, educational and economic impact	(M3)*4/(N5)*4/(N6)*5/(L2)*2/(H2)*2/(H5)*1
	A call to duty	My responsibility	(M1)*14/(M2)*5/(M3)*6/(M4)*1/(M5)*3/(N1)*12/(N2)*4/(N3)*5/(N4)*5/(N5)*7/(N6)*3/(L1)*1/(L3)*9/(H1)*4/(H2)*3/(H3)*5
		An honour to serve	(M2)*1/(N2)*1/(N4)*2
		Feeling skeptical	(M4)*2
		Loss of clients	(M3)*6
		Fighting the challenge	(M1)*2/(M2)*3/(M3)*2/(M4)*1/(N1)*1/(H1)*2/ (H3)*1
	Treatment of FHCWs	Government need to do more	(M1)*10/(M2)*8/(M5)*1/(N2)*2/(N6)*3
		Kudos to hospital management/ partners	(M1)*1 /(M2)*7
Working with COVID-19 patients	Taking care of patients	Excited working with patients	(M2)*4/(M4)*3/(M5)*3/(N1)*3/(N2)*2/(L1)*5/(L2)*5/(H2)*3
		Caring for patients	(M2)*2/(N3)*18/(N4)*9/(N5)*9/(N6)*10/(L1)*5/(L2)*2/(L3)*6/(L4)*1/(H3)*4/(H4)*2/(H5)*4
	Types of patients	Difficult patients	(M3)*6
		Accommodating patients	(M3)*8
		Difficulty in accepting status	(N4)*7
	Enormous task	Enormous task	(M1)*4/(M3)*5/(L1)*2/(L3)*6/(L4)*1/(H2)*5/(H5)*2
Psychological, mental and emotional trauma	Psychological and emotional trauma	Feeling tired, discouraged and depressed	(M1)*7/(M3)*5(M4)*5/(M5)*2/(N1)*4/(N5)*2
		Fear of infection	(M4)*8/(N4)*7/(H5)*1/(L2)*2/(L3)*1
	Mental challenge	Cutoff of relationships	(M2)*4/ (N2)*1/(N3)*5/(N4)*5
	Stress	Fever experience	(L3)*5/(L4)*1
		PPE stress	(N6)*2
	Priming the mind	Living with the pandemic	(L1)*2
Stigmatization	Stigma associated behaviours	Feel stigmatized	(M1)*3/(M2)*13/(M3)*1/(M4)*3/(M5)*4/(N1)*6/(N2)*2/(N3)*2/(N4)*5/(N5)*6/(N6)*3/(L1)*1/(L2)*2/(L3)*1/(L4)*3/(H1)*2/(H2)*5/(H3)*1/(H4)*1/(H5)*3
		Stigmatized by family/friends	(M2)*3/(M3)*3/(M4)*2/(M5)*2/(N2)*2/(N4)*2/(N5)*2/(N6)*3/(L2)*4/(H1)*1/(H5)*1
		Stigmatized by colleagues	(M1)*4/(M2)*1/(M3)*11/(M4)*5/(M5)*3/(N1)*9/(N2)*1/(N3)*8/(N4)*7/(N5)*4/(N6)*5(L1)*3/(L3)*6/(L4)*4/(H1)*3/(H2)*5/(H3)*6/(H4)*4/(H5)*2
		Stigmatized by community	(M1)*3/(M2)*1(M3)*6/(M5)*1/(N1)*3/(N2)*2/(N6)*1/(L1)*4/(L3)*4/(H2)*9/(H3)*4/(H4)*3
	Effects of stigma	Withdrawal behaviour	(M1)*4/(L1)*6/(H2)*2/(H5)*1
	Reasons for stigmatization	Fear of infection	(M1)*8/(M2)*2/(M3)*4/(M4)*3/(M5)*1/(N1)*3/(N3)*4/(N4)*3/(N5)*4/(N6)*1/(L1)*3/(L4)*2/(H1)*1/(H4)*1/(H5)*3
		Limited/lack of knowledge	(M2)*3/(N2)*2/(N3)*6/(L1)*3/(L2)*3/(H3)*5
		Working at the isolation centre	(L3)*2/(L4)*4/(H2)*2/(H5)*1
Recommendations	More knowledge on infection, prevention and control	Education and awareness creation	(M1)*1/(M2)*4/(M3)*12/(M4)*6/(M5)*2/(N1)*2/(N4)*8/(N5)*2/(N6)*1/(L2)*7/(L4)*1/(H2)*2
		Observation of preventive measures	(N5)*3/(N6)*3
	Improvement of services of FHCWs	Motivation of FHCWs	(N4)*8/(N3)*1/(L1)*2/(H1)*1/(H3)*4/(H5)*5
		Need for more laboratory equipment’s	(L3)*1/(H3)*1
		Increase manpower	(N3)*1
		Health insurance	(M1)*1/(M2)*1/(M3)*4/(M4)*2/(M5)*2/(N1)*4/(N2)*1/(N3)*1/(N4)*1/(N5)*1/(N6)*1/(L1)*1/(L2)*2/(L3)*1/(L4)*1/(H1)*1/(H2)*1/(H3)*1/(H4)*1/(H5)*1

#### Subtheme: effects of the pandemic

The effects of the pandemic expressed by the respondents were; repatriating of foreign citizens’, worries about the country’s level of preparedness, skepticism about the future of the country, anxiety, public fright and high impact on life; economic and social aspect (Table 2). Others view the situation as a professional responsibility and the need for the government to step up its support in fighting the pandemic (Table 2). Some participants narrated their experience as follows.

“In February, we had people who were suspect cases but were never confirmed. By March, there was anxiety all around, there were a lot of uncertainties too; people didn’t know what to expect, people were not sure, there were lots of predictions with regards to Africa, lots of predictions with regards to Nigeria. We saw foreign countries repatriating back their citizens, there was a lot of fear and anxiety in the air. For us, it was not any different because we knew we were going to face those cases when they come over. On our path too, there was a lot of fear because this is the first time we are experiencing a pandemic like this. We were also worried about our preparedness; can we sustain the tempo? can we sustain the esteem? All that we heard of in terms of protection; are we going to receive protection? Was it going to be there for us all the time? Our families? So? Yeah! A whole lot! It was climaxed with lots of uncertainties but we were just looking forward to what the deeds are going to unfold in the future” (M3).

“It is worldwide, it is global and cuts across every aspect not only in terms of education. It has put the whole country in a standstill, and is one of those things that I have come across that everybody’s hands just have to be on desk to ensure that we curtail the disease” (M4).

“It is a global emergency; a new disease that just came out which we have not really understood it very well and the impact on lives, economies and social aspect is much. It is something that has no regard for any nation whether developed or under developed and requires prompt attention because of the highly infectious nature of the disease. We are equally privileged; we are among the frontline health workers that are battling with the pandemic. Although the task is much, but with equipment and dedication we are winning the battle” (N5).

“It’s a novel disease that we are not familiar with and because of that, when it came in, there was so much fear when we started seeing it in other countries, so that fear has been in us, in our families, until when it came into the country, and with the casualties we are seeing in other countries, we were also having that fear that it might also happen in our country” (L2).

“When it came it was scary, even now, though we are not that scared, we are very much careful with what we do. The pandemic is seriously affecting people’s lives and we have to be careful about it and know how to go about doing our jobs” (H5).

#### Subtheme: a call to duty

The FHCW felt that it was their duty to engage in the care and treatment of patients. Some saw it as a privilege and honour to serve their country and a challenge that needs to be addressed (Table 2). A respondent was skeptical on accepting the task while another respondent expressed disappointment in loosing of clients on accepting the task (Table 2). Some of their responses is narrated as follows.

“Well, the task is on infection and my specialty is in infectious diseases, so, it’s something I wouldn’t mind on me and I had to participate. Though it was said that it’s optional, but it will not be nice for me to reject the offer. It is something that you have to do if it comes to your field of profession. So, I didn’t feel anything negative about it, it’s okay” (M1).

“I felt it was something I had to do being an infectious disease physician and we are dealing with an infectious disease. So I felt it was my duty to be part of the team even though I was not forced to be part of it, I accepted the challenge. Of course there was this fear of the unknown, we were dealing with the pathogen we have never seen before and there was a lot of fear associated with the virus. I was afraid that I might get infected along the way but even with that I felt it was my duty to be part of the team” (M2).

“For me it wasn’t difficult because of the initial reservations I had; I really didn’t think it was going to come, I thought it was going to be like what we had in previous epidemics that occurred in which we were spared. I thought it was going to be the same kind of setting but certainly I knew if called upon, I was going to respond. By virtue of training, I am a respiratory physician, I knew very well that COVID affects the respiratory system. Is like having a military general who is asked to go to war and he says, he is not going because of fear or something. For me, even though there were uncertainties, I was looking forward to the challenge. There aren’t much reservations really. From the beginning, I have calculated the risk and I just felt that it was worthwhile accepting the task. For me, it was more of if I could just make an impact where I can save people. I wasn’t even bordered about what the outcome for me was going to be. It was a challenge I picked from the beginning and I was looking forward to doing it” (M3).

“Well, I see it as a good experience. Yes, I was brought in by the management and initially I felt skeptical because I felt I am a gyneacologist, so, what am I doing with infectious disease but considering the fact that nobody is immune to this disease, everybody can be exposed, we had pregnant women, we had some postpartum and antenatal patients that have been exposed to COVID. So, I see it as a wonderful experience when I came in, I discovered though we had less than ten of such patients since inception but it’s a wonderful experience and I picked up the challenge” (M4).

“I didn’t think twice because somebody has to do the task and the knowledge that God has deposited on me is not for the fun of it and failure to use that knowledge is also a sin in the sight of God. I have been endowed with the knowledge and I should be able to use it to save others” (N1).

“Well, I feel it is an honour and a responsibility to the profession, to the country and generality of humanity at large” (N2).

“Well, as someone who specializes in microbiology and biomedical virology, I feel it’s a challenge to me to go in there and see how we will fight it out because that’s my area of specialization” (L1)

#### Subtheme: treatment of FHCW

Participants expressed the need for the government to show concern to the FHCW; the need for emotional support and motivation (Table 2). They also expressed the commitment of the hospital management in meeting the needs of FHCW (Table 2).

I am exhausted, I feel frustrated and sometimes I feel like I should dump the job. In fact, I feel neglected, I feel there is nobody apart from my friends that has ever bothered to enquire about how stressful it has been or how I cope with the task. Nobody has done that at all levels, in fact, people try as much as possible to evade or to pretend not to notice so that they don’t give me an opportunity to even talk about it, to bring up a topic or issues that may disrupt the work, but if you need the work to operate smoothly, I feel engaging and interacting with the staff is essential. As I have said, from government downwards this is what I have experienced; in fact, the only thing sometimes you get from government, is not that is official, something perhaps I cannot confirm and cannot say with degree of certainty is, that there are perhaps certain threats that you must do this, it’s your work. Yes, it’s my work but not to the extent of just risking my life. I feel that some people feel that they are at the realm of affairs, at the top and have never bothered to even visit from a distance, to say, that is an isolation centre and wave to the staff and enquire; how are you? They have not done that and have severed themselves from us based on my understanding-” (M1).

“From the hospital management, our management has been supportive in trying to meet our needs, the needs of the frontline healthcare workers working at the isolation centre. From the side of the government, they have provided some support such as the materials that we are working with like the PPE, consumables and all that; we’ve had support and we have not lacked materials to work with. Regarding emotional support, we have not gotten because nobody from the side of the government has bothered to come and find out what we are doing and what has been our challenges so far. Financially, we’ve not really gotten much support from the federal government, they promised to give us additional 20% of our basic salary to all the frontline health workers, they paid for 2 months and that was it; based on the number of months we have worked here it’s really not encouraging with regards to this aspect, the government has not done well” (M2).

### Theme two: working with COVID-19 patients

There were various experiences with regards to working with the COVID-19 patients as indicated in Table 2.

#### Subtheme: taking care of patients

Participants were excited working with people recovering from COVID-19 which was associated with their healing while taking care of them (Table 2). Some of their experiences is narrated as follows.

“I feel very excited to work with them because I see some of them coming in a very bad state, we commence treatment, they get better and go home. I am very happy because I see them come with a lot of symptoms, we commence treatment and they get better” (M5).

“Oh great! If there is anything that gives me passion is to count myself among the people who give other people hope to live. To me, it’s a privilege” (N1).

“My relationship with the patients has been cordial because the disease can infect anybody, it doesn’t respect who you are, where you are from. So, that they are COVID positive doesn’t mean that I am better than them. I didn’t really feel somehow, all I need to do, is just to put in my best, put on protective measures such as the PPE, well kitted, go and see them, talk to them like we have known each other before and this has given them some form of psychological support. For the patients to leave their houses and come to the isolation centre, the stigmatization they might have experienced in the society and coming to the hospital environment, if the healthcare workers stigmatize the patients, it may deteriorate their condition or psychologically affect them. Mostly, when I go, I relate well with them, talk to them, reassure them patterning their state of health, treat them and most of them feel happy about the care they are getting especially those that came from other hospitals. There was a patient that said; wow! You mean you people can come and talk to me and touch me? She was impressed because from the hospital she was coming from, nobody bothers to come close to them. Here we are, not just coming close to them but on several occasions, we just pat them, like reassurance, saying, don’t worry, you will get well, so, all those things also helped” (N3).

#### Subtheme: types of patients

Some of the patients were described as accommodating while others were difficult to work with (Table 2). Majority of the patients that were admitted in the isolation centre had difficulty in accepting their disease status, they had to be counselled before accepting their disease status (Table 2). Some participants narrated their experiences as follows.

“Ah! That’s a damn lot of issues now. Well, you know, they always come in different forms based on their approach to issues generally. Some do understand with you, so we could categorize the patients. For instance, back then we knew that some had this notion that you are doing a government job; so, their whole attitude was, you just do the job irrespective of the safety measures and mechanisms that we put in place. Sometimes you just see them breaking these rules to our own detriment. I feel the notion in their mind was, it’s your job, you just face it and face your business. Then you have some patients who are very obedient, they keep to the rules, they understand you, appreciate what you are doing, they don’t want you to get infected, they understand you and when you explain to them, they are very forth coming. So, we had a range of patients from those who understood what was going on, to those who have a different attitude towards the services we were rendering. We were the second isolation centre, so when we accepted the task, there wasn’t any health insurance, there was nothing, we just accepted the national assignment. So, when you hear some of the patients talk about us, as if is something that was optional to us, it is a little bit discouraging, you source for strength for yourself and keep pushing” (M3).

“Initially, most of the patients need to be convinced that this virus is real. At first, none of them will believe and tell you that; I tested positive. That is the first difficult task. Eighty percent of them that come, don’t believe that they have the virus. So, as a nurse, the first thing I do is to counsel the patients and inform them on the disease and the mode of transmission and the need for them to accept the test result. The anxiety they have in accepting that they have COVID, is what makes is necessary for us to counsel and educate them, before accepting their disease status and we take them into the ward. The first 12 to 24 hours, there is anxiety, high level of fear and rejection. After 24 hours, they relax their minds. The environment too contributes and you see that interaction with them helps. We take all our patients as family and that is the joy in this place. That family attribute, discussing with them one on one, seeing how we take care of them, talk to them, come close to them; the rejection that they feel from outside is no more there, so they feel at home and that is what gives us our quick recovery of patients in this centre. Most of the patents testify to that (N4).

#### Subtheme: enormous task

Participants expressed fear of being infected and discomfort when wearing PPE for long duration while others described the task as enormous (Table 2). The experiences of some of the participants is narrated as follows.

“It has not been easy because you need to spend much time with most of the patients and with the nature of the disease, you know, we have limited time to spend with them but sometimes we go the extra mile to stay with them because of their condition. Some patients are in severe distress, which requires prompt attention and adequate care, we cannot just neglect them because the disease is deadly, we just have to do our best. When you are in PPE you know, it is usually not comfortable but we are still adapting and doing our best” (N5).

“The task is enormous but because it is what we are ready to do, you know, where there is a will, there is always a way, because we have taken it upon ourselves to do, and we are ready here, it doesn’t matter what it will cost us. It’s just like you have committed yourself and you cannot reverse your decision. In difficult times; you say ah! I have not rested. You know during this COVID pandemic, many of our colleagues in other organizations and all the ministries were resting at home without coming to work, everywhere was shut down, even when there was ease of the lockdown, they come to work once in a while. We are coming every day, 24/7, no weekend, no Saturday, no Sunday, yet we are doing it, believing that God is a rewarder” (L3).

### Theme three: psychological, mental and emotional trauma

Respondents were psychologically, emotionally and mentally challenged while carrying out their duties (Table 2). Some respondents were psychologically traumatized; they felt exhausted, frustrated, neglected, demoralized and depressed. Other challenges include lack of health insurance for FHCW at the isolation centre, discouragement from doing the job by their loved ones and emotionally down due to the death of some of their patients. Some of their struggles were movement restriction, separation from family/friends and cutoff of relationships (Table 2).

#### Subtheme: psychological and emotional trauma

A respondent felt discouraged and withdrawn when his colleagues at the isolation centre tested positive; which made him not to come to the isolation centre for some days (Table 2). A respondent had psychological fear of dealing with a highly infectious virus (Table 2). Some of the participants narrated their experiences as follows.

“Sometimes I get depressed and depression sets in when I see one or two of my colleagues who we started this work with coming down with infection and you would expect that we have a good health insurance coverage that will carter for these people and it’s not just there. The people will have to source for their own treatment themselves and you start thinking, is it really worth all the struggle? So, at some point in time, when I am faced with some of these experiences, it really, weighs me down and I start thinking that maybe I should have a rethink on the whole thing” (M3).

“When we talk of performance, the one that really affected me was when I discovered that seven of my colleagues were infected. We had to admit them and these are my colleagues we were walking with day to day. At that point I felt like, do I still have to continue this work? At that point, a lot of thoughts came to my mind; I felt that if anything should dare happen, who will take care of my family? What will become of my family? At that point I felt withdrawn. I remembered, on a certain day, our General Officer had to send a message to me personally, asking if I am okay? I hope all is well with you? Is just that I felt withdrawn. We have been discharging people and we have seen the success rate. I think the only thing that affected me was the fact that I felt withdrawn and I wished I could be exempted from the work. What I mean by withdrawn, was that completely, I don’t even come to the isolation centre to work and even if they comment on the platform I don’t comment on it; that is for that period. I wished I could go back to my day to day activity, before COVID” (M4).

“Ah! Psychologically you will be demoralized, for the view of the society, my colleagues outside and my loved ones. At the initial stage of COVID-19, I have received call from family and well-wishers; we heard that you have decided to work in the COVID-19 outbreak, this disease that is killing everybody, do you want to kill yourself? All sort of things and it took me a great task to convince them that I am not going there to kill myself but to save lives and someone else has to do the job. When you go to the hospital gate to buy orange, they will ask you, what are you doing here? Are you not supposed to be at the isolation centre with your people? They keep their distance and if someone is talking to you, they will ask him why and inform him that you are working with COVID-19. Psychologically, it is traumatizing. It cannot be over emphasized, I am having a lot of psychological challenge because of doing this job” (N1).

“Yes, there is not much difference, the only difference now is psychologically within you, you are dealing with a highly infectious virus that you cannot see. Then, you don’t even know when you will come in contact with this virus despite the PPE you are wearing. Are you getting this virus when you are duffing? Are you going to come in contact with this virus when you touch the patient or when you interact with the patient? Is your PPE anywhere breached? So, all these things play a role in the sense that you just have to be careful compared to when you are working in the ward you were before coming to work at the isolation centre” (N4).

#### Subtheme: mental challenge

Some of the experiences of participants (Table 2) regarding mental challenge is narrated as follows.

“Mental health has been a big challenge, initially when we started we were not going home to our families, that was a very big issue because staying away from your family for months is actually a very big challenge, for me, that was an issue” (M2).

“The first challenge is being confined to a room and separation from my family**.** The truth about it is when we started, we spent more than 2 or 3 months without seeing our loved ones. From the isolation centre, to our hotel room, from the hotel room back to the isolation centre. The first time I saw my loved ones, everybody was scared of coming close to me because they just believed that if they touch me, they will be infected. That mental ability of curtailing that rejection from my own family and my colleagues at my place of work, it really weighed down on me but with time I picked it up and said these are some of the challenges I have to face; and that gave me courage. Later on, they started coming back to interact with me” (N4).

#### Subtheme: stress

Some of the stress encountered by participants in performing their duty was the log duration in wearing of personal protective equipment (PPE) and fever (Table 2). Narration by some of the participants is presented as follows.

“There is difference because before at our various departments, we don’t need to put on PPE while attending to patients, but now before going into the isolation centre, we have to put on complete PPE which is discomforting; we have to stay like 30 minutes, an hour, two hours, so, it’s really affecting us” (N6).

“When we started about a month or two, it was a kind of fever experience, I don’t know whether the fever was as a result of anxiety, fear or whatever, I really don’t know. Three of us working in the laboratory, had fever and we took medications. We don’t know if it was COVID but we called it fever because none of us tested for COVID. Mentally we assumed that anything can happen since we will be dealing with COVID. A day before I came, there was a consultation I had with heaven and God gave me a verse that settled my mind; lo, I will be with you and nothing will happen. I don’t think of anything outside God who has given me the assurance; I don’t have fear” (L3).

#### Subtheme: priming the mind

A respondent expressed the need to brace up in living with corona virus (Table 2). The participant narration is as follows.

“Well, in my opinion, COVID-19 is something that will not be eliminated easily and we have to just prepare our minds to live with it” (L1).

### Theme four: stigmatization

All respondents felt stigmatized while working at the isolation centre (Table 2).

#### Subtheme: stigma associated behaviours

Participants were stigmatized by family, friends, colleagues and their resident communities (Table 2). Some of their experiences is narrated as follows.

“That’s the part I am asking you, which part of stigma? I think I can write a book about stigma. From the beginning, my colleagues have zeroed us as if we were the corona virus itself. It was like a corona virus moving around. Of course, our colleagues will not associate with us and any sight of us, was like we were transferring the infection, since they viewed us as the virus and we are just coming to infect them. At the early part of the pandemic, we needed to take care of our basic needs too, feeding and all of that. If you need to go out, because you are providing services at the isolation centre, you can’t cook, you can’t do anything. You can’t walk out even to just pick up food from an eatery without people watching and saying, oh! He is here! He is here! At some point in time, I had a terrible experience from a colleague who asked me to leave the eatery. So, from family I understand with them. My colleagues, I try to understand with them because these are hospital healthcare workers who should know better and who should have been in the forefront of educating people but because everybody was afraid, let me just be frank, even they were afraid of COVID. So, we were just restricted in one place. It’s as bad as even buying things outside because when they see you outside, it’s like; oh God! what’s this man doing here?” (M3).

“What made it worse was if my colleagues who should know better treat me like this, how much more of somebody outside who is not a doctor or nurse or who doesn’t know anything about me? That was just the issue. Initially, I discovered that once I enter the department, they call me COVID doctor. If I come, nobody wants to interact with me but now everybody does, probably because time has already taken over and some even come to my office to ask questions on what to do? In the department, we had a suspect case they called me and asked of what they should do. At the early stage there was stigma that nobody wants to interact with you. They run away from you and all that” (M4).

“A lot! Not immediate colleagues here, our colleagues in the Teaching Hospital. A lot! A lot! When you have cause to go and get drugs in the hospital and as you reach there, they will tell you, stop there! Stop there! Don’t touch our things. A lot! It happens often“ (N1).

“Yes! Some of them will tell you that, had I known that you will work at the isolation centre, I would not have consented from the beginning. I had to tell them and educate them that it is not a death warrant, even to the victims as well, there is hope for them. if some of us back out, what of the patients that are out there? Who will be there for them? It could be them, it could be we and if everybody backs out, who is going to do the job? So, sometimes, you have to take responsibility” (N2).

“Yes! Yes! The issue of stigmatization is there; from your family, from your colleagues. When we started, you cannot even cross to the main hospital, everybody will be running away from you. I remembered when I went there to pick something; I was reported straight to my coordinator; they warned me not come close to their unit; I should remain in the isolation centre. Later on, when they found out that the disease was not just community spread but also hospital spread, they felt they were more at risk than those of us working at the isolation centre, so they became friendly. That stigma was there and everybody was scared of people working at the isolation centre” (N4).

“The stigmatization is that they assume you are a corona patient, even within the hospital. In fact, initially when we started working in the laboratory at the isolation centre, there were some things we needed to get from the main laboratory; when they see us coming, our colleagues will run away from us because we work at the corona centre. It is assumed that you are infected and you are coming to infect them. These were the experiences we had at the initial stage. Up till now our colleagues are not comfortable being around us; they always point to the fact that we work at the corona centre, because they know we are working at the corona centre. The fact that we work at the corona centre is a problem” (L3).

“Much! Much! Some people that know that I am working here totally don’t want to associate with me. last week, I went to a compound to fetch water, tap water, in the compound they know I am working here, the owner of the compound, the woman, asked me not to touch her tap, she called her son to come and open the tap for me, the only thing I touched was my gallon that I came with, even her gate, they opened it for me. So, I was embarrassed but I am aware of the issue of stigma in the community as a result of the pandemic” (H2).

“I had challenge with my community. The head of the community met me and told me that since I am working at the isolation centre, I have to leave the community because of the information they heard about the virus. It was something scary and since I am working at the isolation centre, I might get infected with the virus, bring it to the community and spread it. So, they need me to leave the community for the period of time I will be working at the centre and for them to observe the trend of the pandemic. So, I left and I thank God for the management that provided a place for us to stay, that was when I felt relieved” (H3).

#### Subtheme: effects of stigma

Some respondents had to withdraw themselves from family, friends, colleagues and their residential communities due to the ongoing stigmatization of FHCW working at the isolation centre (Table 2). Some of their experiences is narrated as follows.

“When we started even our colleagues that are healthcare workers and are not part of the isolation centre workers do not interact with us. Even now, some don’t feel comfortable interacting with us. So, because of that, it has restricted what I do and where I go. I am withdrawn most times because I don’t want to go to where I know people will stigmatize me because I work at the COVID isolation centre. Some people see us as people who can infect them. I tried not to go to some places so that should there be anybody that gets infected with COVID, they will not subsequently link it to me and blame me as a source of infection to that person. So, the stigma especially from health care workers is there” (M1).

“Even at home, my neighbour since he knew that I work at the isolation centre, stopped my children and grandchildren from going to his house and stopped his family from coming to my house. Some people from my state and people I know very well that used to come to my house stopped coming because I work in the corona virus centre. I think I am viewed as infected or possibly carrying the virus on my body, I don’t know. I stopped visiting, I don’t go to people’s houses, I manage to go to church and I have a corner I sit, I don’t stand up to go to places in the church, once the service is over, I go straight to my car, wait for my family members and drive away“ (L1).

#### Subtheme: reasons for stigmatization

Reasons for stigmatization narrated by some of the respondents ranges from fear of getting infected, limited or the lack of knowledge and working at the isolation centre which has to do with treating and taking care of people recovering from COVID-19 (Table 2). Some of the respondents that stated fear of infection as one of the reasons for stigmatization narrated their experiences as follows.

“It’s fear; fear of getting infected. They are afraid of getting infected and they know that this infection doesn’t have a cure. So once they get infected, they are thinking that their chances of dying are high. So, as much as they can, they should avoid anything or anybody that can expose them to the infection; health care workers at the isolation centre, have a high chance of transmitting infection or getting infected if there is any breach in the COVID protocol” (M1).

“I think it is just the fear, it’s better now. Back then at the onset, with the prediction and what was happening, thousands were dying globally, we were seeing thousands needing ventilators. So, of course, every other person around too was afraid. If I come down with COVID, there is some uncertainty; will I survive? will I die? The real issue will have been the fear factor; people were not sure of what to expect” (M3).

“I guess is because of the fear of the disease, the high infective rate of the disease and the possibility of death. Nobody wants to die, because they think by going closer to them, I will infect them. They felt that if I am going inside the isolation centre, I may be a source of infection to them” (M4).

“Of course, I think I know; one is because everybody is scared and they think that once you get the disease, it’s a death sentence. If you are coming close to them, they think you are coming with the disease not knowing that with the knowledge you have gathered, you know all the necessary percussions to take. They even think that when you talk to somebody on phone you will be infected. Your coming, they see you as a danger to their own health. I think there is need for more sensitization about the disease. They should know that there are precautions you can take while you are in the mist of other people” (N1).

“Because of the deadly nature of the virus, people are scared, they know that once they come close to you, or you come close to them they will get the virus if you are infected. That is why the people are scared but now that they have been enlightened, the level of stigmatization has reduced” (N5).

“Of course you know as a pandemic, everybody is afraid, so that he or she will not fall victim. Immediately they say we are the frontline health workers working at the isolation centre here; any time they see us, they don’t want to come close to us, they will be running away from us, despite wearing of facemask, they are still running away from us” (L4).

“Uhmm! The reason people are scared is because they know that I work here. They think I must have been infected and since they think the disease is air borne, by going close to them, they will become infected. Now, I think the scare has reduced” (H5).

Some of the respondents attributed limited knowledge of corona virus (Table 2) as the reason for their stigmatization and narrated their thoughts as follows.

“Lack of adequate knowledge about the disease, because we should not be people that are stigmatized but people that are celebrated, at least for being a frontline health worker. So, I think proper education will go a long way in stopping all these stigmas” (M5).

“It’s due to ignorance; when you have professional colleagues and they are running away, they have fear, it is ignorance. When you have people that don’t have medical knowledge; those ones can run away because of general fear” (L1).

“Lack of education; when people are not educated, they don’t even know how to go about it, the implications and all that. So, they will stigmatize you because they feel you are carrying the virus on your body and you are coming to transmit it to them. If they are enlightened, trained and educated on that, both in the community and their working place, they will not stigmatize you” (L2).

“Lack of knowledge! Lack of knowledge! At first when the disease came, it was not well known, the knowledge was not vast. So, their ignorant in that aspect, if they have orientation, I think it will help a lot” (H3).

Some respondents stated that working at the isolation centre and public misinformation (Table 2) was one of the reasons for the stigmatization and narrated their feelings as follows.

“It’s simply because I am working with the corona patients and the disease is contagious, you can be infected through the air or through hand shake. All of these is what is responsible for the stigmatization” (L3).

“It is because they know I am working at the isolation centre. Those that know that I work here are the ones keeping their distance. Like in my compound, my next door neighbour at first she does the same thing to me but I made her understand that is not what people are saying or thinking; if you follow the social distancing rule, waring facemask and disinfecting your hand, you can relate with them. It is not a killer disease, even if you are infected, by the grace of God, you will get back on your feet, if you follow the prescription” (H2).

### Theme five: recommendations

Some recommendations made by respondents were public sensitization and improvement of services of FHCW (Table 2).

#### Subtheme: more knowledge on infection, prevention and control

Education and awareness creation along with the observation of preventive measures were recommended by respondents as part of measures in curtailing the spread of the outbreak (Table 2). Some of the respondents’ narrations is as follows.

“More education about the virus; creating awareness among the populace will help to reduce the stigma. Letting people know that the fact that someone is working in the isolation centre does not mean that he/she is having the virus all over their bodies. We need to educate people to understand that we are human beings and we need their support. If everybody runs away from us, it will be very traumatizing for us. We are doing something that is honourable and we expect the people to appreciate what we are doing and not to stigmatize us. I think with more understanding and more awareness education, the stigma will reduce” (M2).

“Education; in my own opinion I think that at some point in time, the information that was coming out was not synchronized because you have on one end government have an idea of what they want to do, but the end users; the message wasn’t passed in the same magnitude, in the same perspective that it should. You find government say you need to use a face mask and the person says why should I use a facemask? Government will say using a facemask will protect you and the other person and the man will say that he doesn’t want to protect himself. I think that a lot should have gone into educating and sensitizing people. I think that the religious bodies and a few key people should be making advocacy on what to do. Education was the real missing link. The method of message delivery did not come out in such a way that people could appreciate the magnitude of the problem that we are going through” (M3).

“Proper education of the population starting with the health worker; tell them that this disease is not just infectious because you work at the isolation centre, we wear complete PPE before attending to the patients and we ensure as much as possible not to get infected with the virus. So, proper education to accept everybody and even to see them as heroes instead of stigmatizing them will go a long way in solving it” (M5).

“Enlightenment, you need to enlighten the society on the disease, the mode of transmission and also to make them realize that in many situations someone has to take a bold step to contain the disease. Instead of being stigmatized, they should be praising us like it is done in a civilized society” (N1).

“The most important thing to do is regular hand washing, maintaining social distance, wearing of facemask and enlighten the community and the populace, let them know, that things are not the way they are thinking” (N5).

“There is need for health education, a lot of sensitization needs to be done everywhere; both to health personnel’s and the communities. I think it is also important in the health sector, not only for frontline health workers but for everyone as long as you are in the hospital community. I think it is important for everybody to be trained, so that they can have understanding of the virus and can also be educating people out there. Let people have some basic knowledge about the virus. Community education and sensitization will go a long way, because if people have this understanding, I don’t think there will be much stigmatization” (L2).

#### Subtheme: improvement of services of FHCW

Some respondents expressed the need for more government involvement in the fight against the pandemic, motivation of FHCW and increase of manpower (Table 2).

“Motivation! Motivation! Motivation! Financial motivation, encouragement, honouring people. There are frontline health workers that are supposed to be honoured. Promotions and others as at when due” (N2).

“I know the management are doing their best but maybe they can increase the manpower, so that the time of exposure will not be much on me. if you are many, you know that you will just come and work once or twice and have your rest, you boost your immunity before you come back, that can help. If they can just give stipends as motivation” (N3).

“Everybody needs motivation but just like I told you, we have a wonderful team here; The Chief Medical Director of the Hospital, is also a member of the team, he is the General Officer, we have team lead, we have house manager, in fact, it’s a pleasure working with this wonderful team and we need motivation” (L1).

“For the service, if there are some of the things we need that are supposed to be here which are not, some of the investigations which we need to do because the equipment are not there we couldn’t do them but for the available ones, we are running them. We need the government to supply the needed equipment’s in the laboratory so as to carry out other functions” (L3).

## Discussion

Findings of the e early stage of the pandemic revealed fear and anxiety based on the uncertainties of the outcome of events. This was also witnessed by various countries repatriating back their citizens. Other findings include; FHCWs rising up to the challenges of their duties and working wholeheartedly. Some of them emphasized their faith as their source of strength and courage. They expressed the need for emotional and financial support from the government; the government was lagging in their care. There was a call for the provision of laboratory equipment and improvement of their welfare package. Limited support by government can cause significant burnout and withdrawal among healthcare workers resulting in increased substance dependence behaviors, leading to considerable functional impairment [35]. Accepting the COVID-19 task to a great extent was expressed as a call to duty based on the specialty of the FHCW. They felt it was something they had to do. However, this was not without initial fear and anxiety as stated by some of the respondents.

Working with the COVID-19 patients have been characterized as being stressful due to the possibility of being infected and the prolonged use of PPE when attending to the patients. Despite that, majority of the FHCW expressed their excitement with regards to patients’ recovery and feeling fulfilled in carrying out their duties. A respondent, categorized patients into accommodating and understanding patients and those that are difficult to work with due to their perception that FHCW are performing their duties and they don’t care if they infect them by omission or commission. Reports from China regarding the mental health of medical workers, state that health workers considered it difficult to deal with the dismayed, uncooperative, panic-stricken and stigmatized patients of COVID-19 which may result in apathy and withdrawal among clinicians [36]. Another issue raised by the FHCW was about patients finding it difficult to accept their test result within 12 to 24 h after they were brought to the isolation centre. This type of reaction “denial” is usually common with asymptomatic patients in a study [37] conducted in Lagos, Nigeria.

The COVID-19 task was viewed by majority of the FHCW as an opportunity to serve humanity and the nation. There was also the feeling of fulfilment by these workers in carrying out their tasks. Some of the negative impressions observed by the respondents in carrying out their duties was the issue of stigmatization. Some felt their job was a professional hazard with the possibility of being infected and transferring the infection to their immediate families. Some regarded the task as enormous due to limited staff working at the isolation centre. Proper psychological well-being of the health care personnel’s in this vulnerable time is absolutely essential [38].

Some FHCW felt that working at the isolation centre has not affected their line of duty in any way while others admitted a great difference from the duties they were carrying out in their various departments before being posted to work at the isolation by the hospital management. These changes were expressed as increased work load making the work more stressful, long duration of wearing of PPE, feeling discouraged due to their colleagues becoming infected and the possibility of them becoming infected. Study by Maunder et al. [35] indicates that caring for fellow ill colleagues during the pandemic may increase anxiety of health workers regarding their competence and skills, making them more vulnerable mentally.

Globally, health care workers are increasingly battling against stigmatization and aggression against them [39]. Our findings revealed all respondents felt stigmatized while carrying out their duties; the stigmatization was mostly from their colleagues who were not part of those working at the isolation centre. Stigmatization was also expressed by some of their family members and the communities they lived in. Previous pandemics such as Severe Acute Respiratory Syndrome (SARS) also reported stigmatization of healthcare providers working with SARS-affected patients [40]. Stigmatization of FHCWs in this study is related to the fact that this category of people is at high risk of being infected since they are directly involved with the treatment and care of people recovering from COVID-19 infection. Less stigmatization by the community was observed because most of the respondents stated that the community was not aware of where they worked. A FHCW was sent away from the community he was living in because the community leader came to know that he was working at the isolation centre. Several reports of eviction due to stigmatization of health care workers have been documented during the course of the ongoing COVID-19 pandemic [39, 41, 42].

Our findings on the effects of stigmatization includes psychological trauma, feeling withdrawn and rejection by the public. The withdrawal of these FHCW was to avoid being stigmatized and embarrassed in public while the rejection resulted in severing of relationships with family and friends. Potential drivers of stigma and aggression towards health care workers are the spread of misinformation and rumours through the mass media and social media. Hence, promoting fear, confusion, and ostracizing of health care workers in a frantic attempt by the public to stay safe [39]. Health care workers are targeted because they are seen as COVID-19 carriers [39]. A study in Singapore revealed fear, uncertainty, stigma with associated psychological distress among some of the primary healthcare providers caused by SARS [40]. Stigmatization towards healthcare workers adds to the physical and mental exhaustion that they bear in due course of their duty hours [43]. Common stigmatizing behaviours include isolation within residential communities, local stores, as well as among friends and relatives [44] as expressed by respondents in this study.

Mental challenges mentioned were psychological trauma in the form of feeling frustrated and neglected, feeling demoralized due to stigmatization by family, friends and colleagues. Others include reduced contact with family, depression due to colleagues becoming infected without healthcare insurance in place to take care of them, the fear of possible infection anytime they had body aches or other symptoms, not being able to visit their families at the initial stage of the pandemic and the fear of losing friends. Exposure to people recovering from COVID-19 in hospitals, being quarantined, the death or illness of a relative or friend from COVID-19, and heightened self-perception of danger by the lethality of the virus can all negatively impact the mental well-being of health workers [45, 46]. Separation of health care personnel from family during an infectious disease outbreak may result in enormous emotional toll on them whereas those that perform hospital duties and have to return home every day are at increased risk of developing anxiety with regards to the fear of transmitting the disease to their family members [47]. Also, clinicians may develop a sense of vulnerability due to a lack of definitive therapy, preventive vaccines, uncertain incubation period of the virus and the possibility of asymptomatic transmission [47]. This study revealed that there is no health care insurance for all the FHCW working at the isolation centre. Our finding calls for the urgent attention of the government to provide health care insurance not just for those FHCW working at the isolation centre but for all the FHCW at the forefront of this pandemic.

Reasons for the stigmatization were fear of being infected, limited knowledge of the virus, misinformation of the public and working at the isolation centre. Previous studies have discussed an intense and wide spectrum of psychosocial ramifications that pandemics can inflict on the general population. The mass fear of COVID-19 “corona phobia” [48] due to the unpredictable course of the disease, intolerance of uncertainty, perceived risk of acquiring the infection can generate negative psychological responses including maladaptive behaviors, emotional distress and avoidance reaction among common people [49]. During disease outbreaks, news of the first death, acceleration in number of new cases and expansive media attention can heighten people’s fears, frustrations, helplessness and anxiety over the situation. This results in misplaced health-protective and help seeking behaviors by anxious public that may lead to conflicts between clinicians and patients, which can be harmful to epidemic control programs and hamper social stability [48, 50, 51]. The FHCW need to be appreciated, supported and accepted in their battle against the COVID-19 pandemic.

Stipulated recommendations were education and awareness creation which could be achieved through active engagement of community leaders, social media and other media platforms and religious leaders due to the vital roles they play in the society Others include training of the HCW on COVID-19 IPC, motivation of FHCW by improving their welfare package and recognition of their service to humanity through award presentations. Changing the method of evacuation of people that tested positive to COVID-19 from their homes in order to reduce the stigmatization; this is in coherence with a study [14] on FHCW recovering from COVD-19 infection conducted in Lagos State, Nigeria. People that tested positive to COVID-19 are usually evacuated from their homes using ambulance that has the tag of COVID-19 on it and people in the vicinity usually [14] stigmatize those that test positive to corona virus including their family members. This form of evacuation along with the community testing procedure and the accompanying stigma prevent people from health seeking behaviours. Some respondents suggested faith in God as a means of hope in the face of the pandemic.

The limitation of this study is that the study was conducted in an isolation centre in Abuja, perhaps, there could be varying experiences in other isolation centres in Abuja or elsewhere within the country.

## Conclusion

The study demonstrates the positive and negative experiences of FHCW. Frontline health care workers were stigmatized, psychologically traumatized and are battling to put in their best in the face of the pandemic. There is a need for the government and society to acknowledge and appreciate the efforts of FHCW. There is need to provide health care insurance for FHCW. Public education and sensitization on infection, prevention and control measures for COVID-19 would be relevant to address stigmatization in the society. In addition, adequate mobilization and engagement of community stakeholders such as traditional and religious leaders, and opinion group leaders should be prioritized in ensuring that FHCW do not suffer stigmatization while providing care at COVID-19 isolation centers.

## Data Availability

The datasets used and/or analysed during the current study available from the corresponding author on reasonable request.

## Abbreviations

FCT: Federal Capital Territory
FHCW: Frontline Healthcare Workers
HREC: Health Research Ethics Committee
NCHRE: National Code for Health Research Ethics
PPE: personal protective equipment
UATH: University of Abuja Teaching Hospital
WHO: World Health Organization
